# A systematic review of determinants of sedentary behaviour in youth: a DEDIPAC-study

**DOI:** 10.1186/s12966-015-0291-4

**Published:** 2015-10-09

**Authors:** Annabel S. Stierlin, Sara De Lepeleere, Greet Cardon, Patricia Dargent-Molina, Belinda Hoffmann, Marie H. Murphy, Aileen Kennedy, Grainne O’Donoghue, Sebastien FM Chastin, Marieke De Craemer, on behalf of the DEDIPAC consortium

**Affiliations:** Institute of Epidemiology and Medical Biometry, Ulm University, Ulm, Germany; Section Health Economics and Health Services Research, Department of Psychiatry II, Ulm University, Bezirkskrankenhaus Günzburg, Ulm, Germany; Department of Movement and Sports Sciences, Ghent University, Ghent, Belgium; Univ Paris Descartes, UMRS 1153, F-94807 Villejuif, France; Division of Sports and Rehabilitation Medicine, Department of Medicine II, Ulm University, Ulm, Germany; Sport and Exercise Sciences Research Institute, University of Ulster, Northern Ireland, UK; Centre for Preventive Medicine, Dublin City University, Dublin, Ireland; Institute of Applied Health Research, School of Health and Life Science, Glasgow Caledonian University, Glasgow, Scotland UK; Inserm U1153, ORCHARD, Centre de Recherche Epidémiologie et Statistique Sorbonne Paris Cité (CRESS), Villejuif, F-94807 France

**Keywords:** Children, Adolescents, Youth, Sedentary behaviour, Screen time, Sitting, Determinant

## Abstract

Sedentary behaviour (SB) has emerged as a potential risk factor for metabolic health in youth. Knowledge on the determinants of SB in youth is necessary to inform future intervention development to reduce SB. A systematic review was conducted to identify predictors and determinants of SB in youth. Pubmed, Embase, CINAHL, PsycINFO and Web of Science were searched, limiting to articles in English, published between January 2000 and May 2014. The search strategy was based on four key elements and their synonyms: (a) sedentary behaviour, (b) determinants, (c) types of sedentary behaviours, (d) types of determinants. The full protocol is available from PROSPERO (PROSPERO 2014:CRD42014009823). Cross-sectional studies were excluded. The analysis was guided by the socio-ecological model. 37 studies were selected out of 2654 identified papers from the systematic literature search. Most studies were conducted in Europe (*n* = 13), USA (*n* = 11), and Australia (*n* = 10). The study quality, using the Qualsyst tool, was high with a median of 82 % (IQR: 74–91 %). Multiple potential determinants were studied in only one or two studies. Determinants were found at the individual, interpersonal, environmental and policy level but few studies examined a comprehensive set of factors at different levels of influences. Evidence was found for age being positively associated with total SB, and weight status and baseline assessment of screen time being positively associated with screen time (at follow-up). A higher playground density and a higher availability of play and sports equipment at school were consistently related to an increased total SB, although these consistent findings come from single studies. Evidence was also reported for the presence of safe places to cross roads and lengthening morning and lunch breaks being associated with less total SB. Future interventions to decrease SB levels should especially target children with overweight or obesity and should start at a young age. However, since the relationship of many determinants with SB remains inconsistent, there is still a need for more longitudinal research on determinants of SB in youth.

**Electronic supplementary material:**

The online version of this article (doi:10.1186/s12966-015-0291-4) contains supplementary material, which is available to authorized users.

## Introduction

Although the evidence is still inconsistent [1], high levels of sedentary behaviour (SB) in youth (<18 year) may be associated with cardiometabolic health, poorer mental health and lower bone mineral content [2–10]. Several studies have shown that a lot of children spend most of their time being sedentary. For example, 10–12 year old European children spend approximately 8 h being sedentary during the day [11]. Furthermore, the ENERGY-study showed that European children spent on average more than 2 h/day in front of screens (TV and computer activities) [12], despite the current guidelines which recommend ≤2 h/day of recreational screen time [13]. A narrative review on SB in adolescents reported that screen-based behaviour ranges from 2 to 4 h per day and total SB ranged from 5 to 10 h per day [14]. Additionally, there is evidence that SB tracks from childhood into adulthood [15, 16], and the evidence for ill health effects of SB among adults is strong [17]. This highlights the importance of youth as an important life stage for addressing SB.

Several interventions to decrease children’s sedentary time have been carried out, but most effects were small [18, 19]. Information on the association between specific determinants and SB, together with the modifiability of those determinants, could guide and inform future interventions targeting SB in youth. To structure the study of these determinants, the socio-ecological model can be used, which places the individual within an ecosystem [17, 20]. Furthermore, the review by Uijtdewilligen et al. (2011), which investigated the determinants of physical activity and sedentary behaviour in young people (4–18 years old), found insufficient evidence for determinants of sedentary behaviour [21]. Additionally, to date there is no summary of available evidence about the determinants of SB in youth that spans the whole age range of 0 to 18 years based on this socio-ecological model. Therefore, the aim of this study is to systematically review the literature regarding potential determinants of SB in children under the age of 18 within a social-ecological perspective. This systematic review is one of three reviews (one in youth (<18 years old), one in adults (18–65 years old) and one in older adults (>65 years old)) performed as part of the DEDIPAC (DEterminants of DIet and Physical ACtivity) study [22].

## Review

### Methods

A common protocol for the three DEDIPAC systematic literature reviews across the life course (youth, adults, older adults) was developed and is available from PROSPERO (PROSPERO 2014:CRD42014009823).

### Search strategy

A systematic literature search was conducted in five electronic databases (Pubmed, Embase, CINAHL with full text, PsycINFO and Web of Science) to detect studies investigating determinants of SB in youth (<18 year old) published between January 2000 and May 2014.

The search strategy was based on four key elements (see Additional file 1): (a) SB and its synonyms (e.g. sedentariness); (b) determinants and its synonyms (e.g. correlates, factors); (c) types of SB (e.g. TV viewing, gaming); and (d) possible determinants of SB (e.g. environmental, behavioural). Terms referring to these four elements were used as MESH-headings and title or abstract words in all databases. The initial search was performed by one researcher (GOD) familiar with the principles of systematic reviewing and searching bibliographic databases for this purpose. Details of the search strategy are shown in Additional file 1. After running the search strategy in each database, duplicates were identified and removed. Two independent reviewers (AS and SDL) screened studies by title and abstract to determine their eligibility for inclusion. In case of disagreement, a third reviewer (AK) was asked to reach a decision. Full texts were divided equally and screened by one of two researchers (AS and SDL). In addition, other experts in this research area were contacted to identify additional relevant determinant studies (e.g. articles from the author group working on determinants of SB in adults which appeared to belong to the children’s results) and backward reference tracking was undertaken for the included articles (MDC). Articles obtained this way were subjected to the same selection process as the articles found initially. Two authors (AS and SDL) extracted data independently and subsequently, three reviewers (AS, SDL and BH) undertook cross checking and harmonisation of extracted data. Discrepancies were resolved through discussion.

### Selection of studies

The literature search was limited to articles published in English. Reviews, editorials, commentaries, letters to the editor, personal views, conference papers, protocols, multi-component intervention studies, and studies focusing on patient groups, were excluded. Furthermore, studies with only cross-sectional analyses were excluded since they do only provide information on association, and not on prediction or causation [23]. To be eligible for inclusion, studies had to meet the following criteria. Firstly, studies had to investigate at least one possible determinant of SB. Secondly, the mean age of the study sample at follow-up had to be lower than 18 years. Thirdly, studies were included if they assessed (1) total SB time, or (2) subdomains of SB such as time spent watching TV, screen time, homework, reading, etc. Studies using subjectively (e.g. questionnaire) and objectively (e.g. accelerometry) measured SB were included (cut off point for accelerometry determined SB: <100 counts per minute (CPM) [24]).

### Data extraction

A standardized template was used to extract data from the included studies using the following headings: general information, sample characteristics, study characteristics, outcome measures, determinants, statistical analysis, results and general findings/comments. The data extraction tool was based on the recommendations from ‘the Centre for Reviews and Dissemination guidance handbook for undertaking systemic literature review in healthcare’ [25].

### Association and classification of determinants

When specific age groups were studied, youth was categorized as follows (1) toddlers and preschoolers (0–5 years old), (2) primary schoolchildren (6–12 years old), and (3) adolescents (13–17 years old). The determinants of SB were classified across four levels using the social-ecological framework applied by Sallis et al. (2008) [20] (i) individual (biological/genetic, psychological/behavioural); (ii) interpersonal (social, cultural), (iii) environmental (micro, macro) and (iv) policy (industry, government).

To determine the consistency of association of each determinant with either total SB or screen time, the model used by Sallis et al. (2000) [26], was applied (see Table 1). In this model, the consistency regarding the association of a determinant with SB is based on the percentage of reported findings that support the hypothesized association measured by the number of findings supporting the association divided by the total number of findings where the association was mentioned. The result was defined as ‘no evidence’ (coded with a ‘0’) if the percentage of the findings supporting the association was between 0 and 33 %; as ‘inconsistent evidence’ (coded with a ‘?’) if the percentage of the findings supporting the association was between 34 and 59 %; and as a ‘consistent association’ (coded with a ‘+’ or ‘-’) if the percentage of the findings supporting the hypothesized association was between 60 and 100 %. In addition, when four or more studies supported the association, the result was coded as ‘++’ or ‘- -’; and when four or more studies failed to show an association, the result was coded as ‘00’.

**Table 1:** Rules for classifying determinants regarding the association with SB (based on [26])

Proportion of analyses supporting the association (%)	Summary code	Meaning of code
0–33	0	No evidence
34–59	?	Inconsistent evidence
60–100	+ /−	Consistent association

When four or more studies supported an association or no association, it was coded as + +, − − or 00

### Risk of bias

To assess the risk of bias, the quality assessment tool ‘QUALSYST’ from the “Standard Quality Assessment Criteria for Evaluating Primary Research Papers from a Variety of Fields” (Alberta Heritage Foundation for Medical Research) was applied [27]. With this pragmatic tool, 14 items of each quantitative study, were scored on the study and outcome levels depending on the degree to which the specific criteria were met or reported (“yes” = 2, “partial” = 1, “no” = 0). Items not applicable to a particular study design were marked “n/a” and were excluded from the calculation of the summary score. A percentage was calculated for each paper by dividing the total sum score obtained across rated items by the total possible score (see Additional file 2). The quality of the included articles was assessed by two independent reviewers (AS and SDL). In case of disagreement, the two reviewers discussed quality scores until agreement was reached.

## Results

**Fig. 1:**
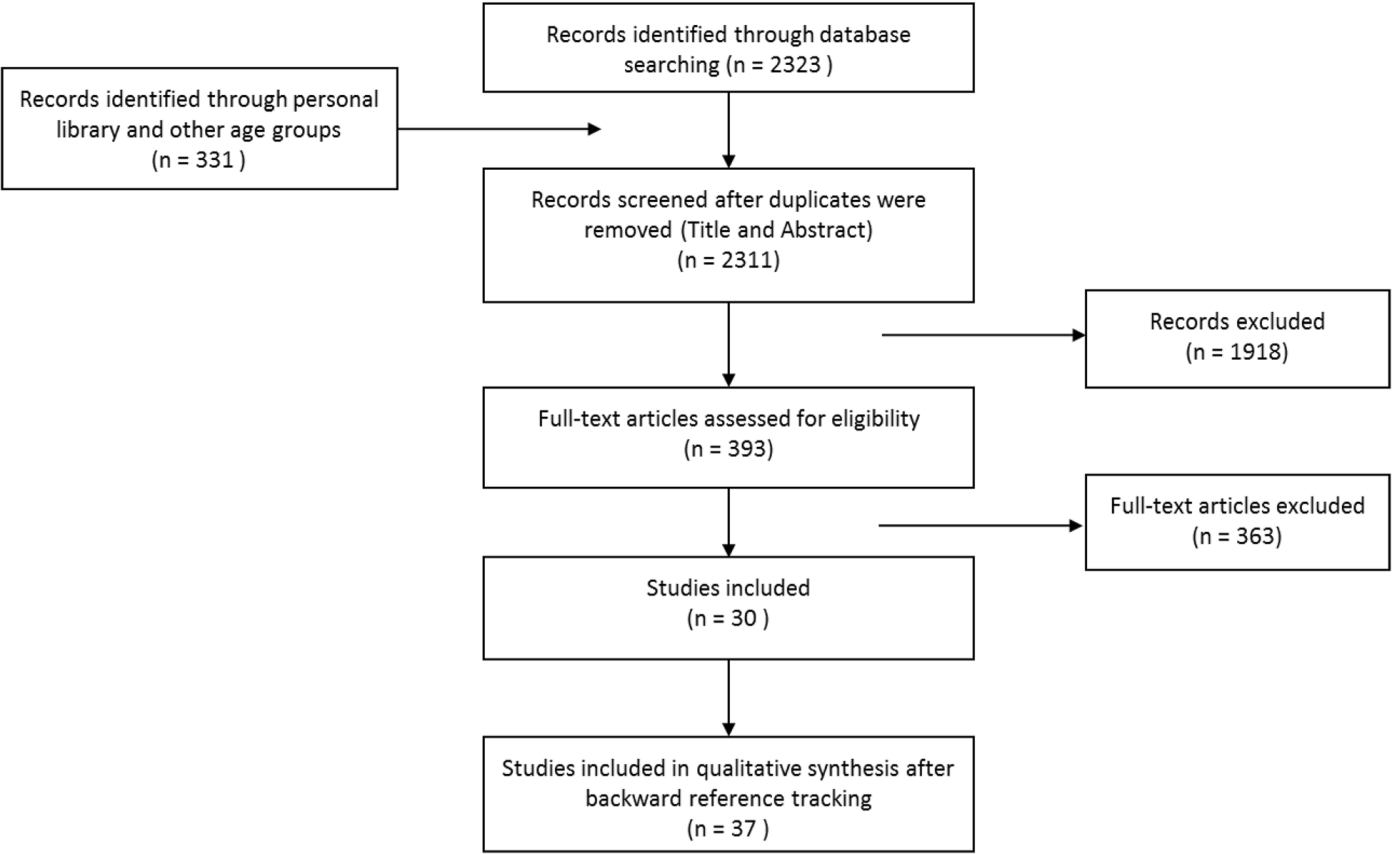
Flow chart of the literature search

The database search resulted in the selection of 2323 articles. Furthermore, 327 extra articles were received from the literature search of the other age groups which were wrongly classified. Three extra articles were added from personal bibliographies. Of these 2654 articles, 343 duplicates were removed. Title and abstract screening of the remaining 2311 articles were screened and resulted in the full texts screening of 393 articles. From these, 30 studies met the inclusion criteria. Backward reference tracking of these 30 studies resulted in the selection of 26 more articles of which seven were included. In total, the review comprises 37 articles (see flow chart in Fig. 1). In Table 2, an overview of the included studies is presented.

**Table 2:** Descriptive characteristics of the included articles

Age group	Author (year)	Country	Design	Participants			Sedentary Behaviour Measure		Quality Score (%)
				Total	Proportion (male/female)	Mean Age in years	General	Specific	
Toddlers and preschoolers	Taylor et al. 2009 [62]	New Zealand	Longitudinal cohort	244	56 % M 44 % F	5 year	Parent-report questionnaire	Sedentary time and screen time	77.3
Children	Telford et al. 2013 [53]	Australia	Longitudinal cohort	853	51 % M 49 % F	12 year	Accelerometer	Sedentary time	95.5
	Atkin et al. 2013b [38]	UK	Longitudinal cohort	854	42 % M 58 % F	11.2 year		Sedentary time	90.9
	Mantjes et al. 2012 [33]	UK	Longitudinal cohort	839	42 % M 58 % F	11.2 year		Sedentary time	90.9
	D’Haese et al. 2013 [36]	Belgium	Cross-over study	187	52 % M 48 % F	10.4 year		Sedentary time	75.0
	Cui et al. 2011 [60]	China	Nested cohort study	1997: 2469	1997:		Self-report questionnaire	TV/video/DVD viewing, video games playing, computer time, homework, reading, writing and drawing	77.3
				2000: 1838	52 % M 48 % F 2000:	11.7 year			
				2004: 1382	54 % M 46 % F	12.0 year			
					2004:				
				2006: 1128	53 % M 47 % F	12.0 year			
					2006:				
					53 % M 47 % F	11.7 year			
	Ziviani et al. 2008 [59]	Australia	Nested cohort study	59	44 % M 56 % F	8.9 year	Parent-report questionnaire	Screen time, homework, reading, musical/cultural activity, craft activity, indoor play, daily care activity	54.5
	Treuth et al. 2004 [40]	USA	Longitudinal cohort	91	100 % F	10 year		TV viewing	63.6
	Davison et al. 2005 [41]	USA	Longitudinal cohort	173	100 % F	11 year		TV viewing	77.3
	Barkley et al. 2012 [45]	USA	Cross-over study	19	58 % M 42 % F	11.3 year (M)	Observation	Sedentary time	67.9
						11.5 year (F)			
	Fuller-Tyszkiewicz et al. 2012 [52]	Australia	Longitudinal cohort	9064	51 % M 49 % F	Cohort K: 6.3 year Cohort B: 10.3 year	Interview	TV viewing	63.6
	Wickel et al. 2013 [34]	Netherlands	Longitudinal cohort	886	50 % M 50 % F	11 year		Sedentary time, screen time, and non-screen time	72.7
	Janz et al. 2005 [15]	USA	Longitudinal cohort	378	47 % M 53 % F	8.6 year	Accelerometer + Parent-report questionnaire	Sedentary time + TV viewing and video games playing	77.3
	Veitch et al. 2011 [51]	Australia	Longitudinal cohort	171	54 % M 46 % F	11.1 year		Sedentary time + screen time, computer/e-games time	81.8
	Hjorth et al. 2013 [27]	Denmark	Cross-over study	785	52 % M 48 % F	10.5 year (M) 10.4 year (F)		Sedentary time + screen time	95.5
	Straker et al. 2013 [55]	Australia	Cross-over study	56	48 % M 52 % F	11.8 year	Accelerometer + Diary	Sedentary time + sedentary leisure time (total, screen, non-screen) and TV/non-game computer time	84.6
	Atlantis et al. 2008 [57]	Australia	RCT^a^	30	77 % M 23 % F	10–12 year	Interview + Observation	Sedentary time	69.2
Adolescents	Evenson et al. 2010 [44]	USA	RCT	847	100 % F	13.9 year	Accelerometer	Sedentary time	86.4
	Ridgers et al. 2013 [54]	Australia	Longitudinal cohort	111	51 % M 49 % F	17.6 year		Sedentary time	86.4
	Ortega et al. 2013 [35]	Estonia, Sweden	Combined analysis of two mixed-longitudinal cohort studies	Swedish cohort: 753	Swedish cohort: 45 % M 55 % F	Swedish young cohort: 15.5 year (Other cohorts are >18 year at follow up)		Sedentary time	90.9
				Estonian cohort: 813	Estonian cohort: 46 % M 54 % F				
	Bauer et al. 2008 [43]	USA	Longitudinal cohort	2516	45 % M 55 % F	Cohort 1: 17.2 year (cohort 2: > 18 year)	Self-report questionnaire	TV/video viewing	81.8
	Brodersen et al. 2007 [32]	UK	Longitudinal cohort	5287	49 % M 51 % F	15–16 year		TV viewing and video games playing	81.8
	Delmas et al. 2007 [30]	France	RCT	379	51 % M 49 % F	15.7 year		TV/video viewing and reading time	86.4
	Hardy et al. 2007 [58]	Australia	Longitudinal cohort	163	100 % F	14.9 year		Sedentary time and sedentary behaviours	86.4
	Nelson et al. 2006 [42]	USA	Longitudinal cohort	2516	cohort 1: 45 % M 55%F	15–18 year (cohort 1)		TV/video viewing and leisure-time computer use	86.4
					cohort 2: 45 % M 55 % F				
	Van Jaarsveld et al. 2007 [31]	UK	Longitudinal cohort	5229	57 % M 43 % F	15–16 year		TV/video viewing, video games playing on computer	90.9
	Schmitz et al. 2002 [47]	USA	RCT	3798	52 % M 48 % F	13.3 year		Sedentary leisure habits	95.5
	Datar et al. 2012 [46]	USA	Longitudinal cohort	18,900	51 % M 49 % F	14.2 year	Parent-report questionnaire	TV viewing	81.8
	Saelens et al. 2002 [48]	USA	Longitudinal cohort	169	52 % M 48 % F	12.1 year	Interview	TV time	72.7
	Raudsepp et al. 2008 [39]	Estonia	Longitudinal cohort	345	51 % M 49 % F	14 year	3-day recall	Sedentary time	68.2
	Atkin et al. 2013a [37]	UK	Longitudinal cohort	sedentary time: 319 screen time: 373	T0 (accel.): 45 % M 55 % F	14.3 year	Accelerometer + Self-report questionnaire	Sedentary time + Screen-time	77.3
					T4 (accel.): 48 % M 52 % F				
					T0 (quest.): 44 % M 56 % F				
					T4 (quest.): 45 % M 55 % F				
	Hume et al. 2011 [50]	Australia	Longitudinal cohort	155	40 % M 60 % F	16.4 year (M)		Sedentary time + TV/video/DVD viewing	81.8
						16.2 year (F)			
	Trang et al. 2013 [61]	Vietnam	Longitudinal cohort	759	48 % M 52 % F	15.8 year		Sedentary time + Screen time	90.9
Children + Adolescents	Arundell et al. 2013 [56]	Australia	Longitudinal cohort	2053	Younger: 52 % M 48 % F	10–11 year	Accelerometer	Sedentary time	90.9
					Older: 45 % M 55 % F	15–17 year			
	Ridgway et al. 2011 [28]	Denmark	Secondary data analyses on four cohort studies	4170	EYHS: 47 % M 53 % F	12.0 year		Sedentary time	95.5
		Norway							
		Portugal			Roots study: 44 % M 56 % F	14.5 year			
		Estonia							
		UK							
					Speedy study: 44 % M 56 % F	10.2 year			
		Brazil							
					Pelotas: 52 % M 48 % F	13.3 year			
	Francis et al. 2011 [49]	USA	Longitudinal cohort	434	47 % M 53 % F	13 years	Parent-report questionnaire	TV time, video game time	90.9
	Murdey et al. 2005 [29]	UK	Longitudinal cohort	83	52 % M 48 % F	Cohort 1: 12.1 year	Diaries	Sedentary time	59.1
						Cohort 2: 14.2 year			
						Cohort 3: 16.0 year			

^a^Data used of the four RCTs that were included:

-Delmas et al. [31]: Only the data from the control group were reported in the manuscript and therefore only those data were used in the review

-Evenson et al. [45]: In each analysis model, the treatment condition (intervention vs. control) was included as a covariate. Therefore, both intervention and control group data could be used

-Atlantis et al. [58]: no significant effects or trends were seen for any of the dependent variables. Therefore, data of both intervention and control groups were used

-Schmitz et al. [48]: The self-reported PA and SLH were measured in spring whereas demographic and psychosocial variables were measured the previous fall (baseline data). Since the 16 schools of this study were randomized to intervention or comparison (delayed intervention) conditions after all baseline measures were taken, both intervention and control group data could be used for the current review

### Study characteristics

Of the 37 included studies, 13 were conducted in Europe [28–40] (of which six in the UK [30, 32–34, 38, 39]), 11 in the USA [15, 41–50], 10 in Australia [51–60], two in Asia [61, 62] and one in New-Zealand [63]. More than half of the studies (*n* = 21) were published from 2010 onwards [28, 29, 34–39, 45–47, 50–57, 61, 62], with 11 in 2013 [28, 35–39, 54–57, 62]. Nine studies exclusively used objective measures of SB by means of accelerometers [29, 34, 36, 37, 39, 45, 54, 55, 57], whereas 15 studies exclusively used self-reported or parent-reported SB from questionnaires [30–33, 41–44, 47, 48, 50, 59–61, 63]. Six studies used both accelerometers and questionnaires [15, 28, 38, 51, 52, 62]. Furthermore, two studies used observations [46, 58], three studies used interviews [35, 49, 53], one study used accelerometers combined with self-reported SB from diaries [56] and one study used recalls [40] to assess SB.

The different age groups (according to age at follow-up) studied were: toddlers and preschoolers (0–5 years old) (*n* = 1) [63], children (6–12 years old) (*n* = 16) [15, 28, 34, 35, 37, 39, 41, 42, 46, 52–54, 56, 58, 60, 61], adolescents (13–17 years old) (*n* = 16) [31–33, 36, 38, 40, 43–45, 47–49, 51, 55, 59, 62], or a combination of age groups (*n* = 4) [29, 30, 50, 57]. The sample sizes ranged from 19 to 18,900 participants with a median of 759 participants. Four studies only included female participants [41, 42, 45, 59], whereas 33 studies included both boys and girls [15, 28–40, 43, 44, 46–58, 60–63]. No studies included only boys. In the included articles, the following designs were used: randomized controlled trial (*n* = 4) [31, 45, 48, 58], cross-over study (*n* = 4) [28, 37, 46, 56] or longitudinal cohort study (*n* = 29) [15, 29, 30, 32–36, 38–44, 47, 49–55, 57, 59–63]. A complete overview of the study characteristics is given in Table 2.

### Risk of bias

Overall, the studies were of good quality with a median score of 82 % and an interquartile range of 74 to 91 %. The lowest score was 55 % for Ziviani et al. (2008) [60]. The highest score was 96 % for Hjorth et al. (2013) [28]. Of all the items of the checklist for the assessment of the quality of quantitative studies, item 1 ‘Question/objective sufficiently described?’, item 2 ‘Study design evident and appropriate?’ and item 10 ‘Analytic methods described/justified and appropriate?’ were most frequently reported. Item 11 ‘Some estimate of variance is reported for the main results?’ appeared to be the item most frequently missing.

### Specific outcomes investigated

Associations of potential determinants with objectively and subjectively measured total SB and subjectively measured screen time are given in Tables 3, 4 and 5, respectively. Other SB domains such as reading, writing and drawing were rarely investigated [31, 35, 40, 55, 56, 59–61] and therefore not mentioned in the table nor results’ section.

**Table 3:** Determinants of objectively measured total sedentary behaviour in children and direction and strength of association

	Related to sedentary behaviour		Unrelated to sedentary behaviour	Summary code^1^	
Variables	Reference number	Direction of association	Reference number	*n*/*N* for row (%)^2^	Association (+/−)^3^
Individual variables: biological/genetic					
Gender	54^b^	-		1/1 (100 %)	-
Age (older)	36^b^, 36^g^, 39^b^, 39^g^, 39, 39, 39, 39, 54^b^, 54^g^, 57^b^, 57^g^	+	15, 15	12/14 (86 %)	++
Birth weight			29	0/1 (0 %)	0
SES (high)	39, 39	+		2/2 (100 %)	+
Individual variables: psychological/behavioural					
Depressive symptoms			51^b^, 51^g^	0/2 (0 %)	0
Interpersonal variables: social					
Family influences					
Number of parents living at home			39, 39	0/2 (0 %)	0
Number of siblings	39	-	39	1/2 (50 %)	?
Parental behaviour					
Paternal PA	39^b^	+	39^g^, 39, 39	1/4 (25 %)	0
Paternal TV/computer use (weekdays)			39, 39	0/2 (0 %)	0
Paternal TV/computer use (weekend days)	39	+	39	1/2 (50 %)	?
Maternal PA			39, 39	0/2 (0 %)	0
Maternal TV/computer use (weekdays)			39, 39	0/2 (0 %)	0
Maternal TV/computer use (weekend days)	39	+	39	1/2 (50 %)	?
Family behaviour					
Going to the park as a family	39^b^	-	39^g^, 39	1/3 (33 %)	0
Playing sports as a family	39^b^	-	39^g^, 39	1/3 (33 %)	0
Visiting relatives as a family			39, 39	0/2 (0 %)	0
Reading as a family			39, 39	0/2 (0 %)	0
Watching TV as a family			39, 39	0/2 (0 %)	0
Rules and restrictions					
Bedtime rules			39, 39	0/2 (0 %)	0
Restriction for playing outside	39^g^	+	39^b^, 39	1/3 (33 %)	0
Rules for playing after dark			39, 39	0/2 (0 %)	0
Indoor play rules			39, 39	0/2 (0 %)	0
Restriction for SB			39, 39	0/2 (0 %)	0
Parental perceptions					
Parents believe there is a high crime rate in their neighbourhood			52	0/1 (0 %)	0
Parents consider stranger danger to be a concern			52	0/1 (0 %)	0
Social network					
Social network score			52	0/1 (0 %)	0
Social trust and cohesion score			52	0/1 (0 %)	0
Ostracism (social support)	46, 46, 46, 46	+		4/4 (100 %)	+
Environmental variables					
Home					
Shared bedroom	39	-	39	1/2 (50 %)	?
Electronic games at home	39	-	39	1/2 (50 %)	?
Active games instead of traditional electronic games	56	-	56, 56, 56	1/4 (25 %)	0
Removal of traditional electronic games	56	-	56, 56, 56	1/4 (25 %)	0
Electronic equipment in the bedroom	39, 39	-	38, 38	2/4 (50 %)	?
Computer in the bedroom			38, 38, 38	0/3 (0 %)	0
TV in the bedroom			38, 38, 38	0/3 (0 %)	0
Neighbourhood					
Urbanisation			39, 39	0/2 (0 %)	0
Area-level deprivation			39, 39	0/2 (0 %)	0
Living in a cul-de-sac			39, 39, 52	0/3 (0 %)	0
Neighbourhood play rules			39, 39	0/2 (0 %)	0
Parents are satisfied with quality of parks and playgrounds in their neighbourhood			52	0/1 (0 %)	0
Distance to closest public open space from home			52	0/1 (0 %)	0
Closest park: area of closest park to home			52	0/1 (0 %)	0
Closest park: number of recreational facilities			52	0/1 (0 %)	0
Closest park: number of playgrounds			52	0/1 (0 %)	0
Closest park: number of amenities			52	0/1 (0 %)	0
Closest park: walking paths			52	0/1 (0 %)	0
Closest park: cycling paths			52	0/1 (0 %)	0
Closest park: lighting along paths			52	0/1 (0 %)	0
Closest park: trees providing shade			52	0/1 (0 %)	0
Closest park: water feature			52	0/1 (0 %)	0
Closest park: signage regarding dogs			52	0/1 (0 %)	0
Safety of walking/jogging in the neighbourhood			45^g^	0/1 (0 %)	0
Walkers/bikers on the streets can be easily seen by people at home			45^g^	0/1 (0 %)	0
Much crime in the neighbourhood			45^g^	0/1 (0 %)	0
Good lighting in the streets			45^g^	0/1 (0 %)	0
Much traffic, difficulties to walk			45^g^	0/1 (0 %)	0
Children frequently play outdoors			45^g^	0/1 (0 %)	0
Many interesting things to look at in the neighbourhood			45^g^	0/1 (0 %)	0
Many places to go within easy walking distance of home			45^g^	0/1 (0 %)	0
Sidewalks on most of the streets			45^g^	0/1 (0 %)	0
Bicycle/walking trails			45^g^	0/1 (0 %)	0
Easy access to 14 specified facilities (e.g. basketball court)			45^g^	0/1 (0 %)	0
Difficulties to get home from after-school activity at school			45^g^	0/1 (0 %)	0
Difficulties to get to an after school activity not at school			45^g^	0/1 (0 %)	0
Difficulties to get home from an activity someplace else			45^g^	0/1 (0 %)	0
School					
Location town fringe			34, 34	0/2 (0 %)	0
Location village/hamlet dwelling (urban)	34	+	34	1/2 (50 %)	?
School size (number of pupils in year 4)	34	-	34	1/2 (50 %)	?
School ground supportiveness for PA			34, 34	0/2 (0 %)	0
Aesthetics score			34, 34	0/2 (0 %)	0
Playground area			34, 34	0/2 (0 %)	0
Playground density	37, 37, 37, 37, 37, 37, 37	+	37, 37, 37	7/10 (70 %)	+
Existence of a bike rack			34	0/1 (0 %)	0
Existence of an entrance for pedestrians/cyclists only			34	0/1 (0 %)	0
Walking access supportiveness for PA			34	0/1 (0 %)	0
Cycling access supportiveness for PA	34	-		1/1 (100 %)	-
Existence of gym facility			34	0/1 (0 %)	0
Existence of indoor sports facility			34	0/1 (0 %)	0
Existence of sports field/pitch facility			34	0/1 (0 %)	0
Existence of pool facility			34	0/1 (0 %)	0
Existence of changing facilities	34	+		1/1 (100 %)	+
Existence of play equipment	34	+		1/1 (100 %)	+
Existence of sports equipment	34	+		1/1 (100 %)	+
Use of local park or playground			34	0/1 (0 %)	0
Medium or high quality of sports facilities			34	0/1 (0 %)	0
Physical activity facility supportiveness for PA			34	0/1 (0 %)	0
Other facility supportiveness for PA			34	0/1 (0 %)	0
School neighbourhood					
Existence of heavy traffic			34	0/1 (0 %)	0
Proportion of A-roads			34	0/1 (0 %)	0
Number of traffic accidents per km of road			34	0/1 (0 %)	0
Existence of pathways near school			34	0/1 (0 %)	0
Existence of safe places to cross roads	34	-		1/1 (100 %)	-
Cars drive slowly			34	0/1 (0 %)	0
Streets are safe to walk or ride			34	0/1 (0 %)	0
Easy to get to school by foot			34	0/1 (0 %)	0
Number of PA facilities per km^2^			34	0/1 (0 %)	0
m^2^ verge per m of road			34	0/1 (0 %)	0
Percentage of accessible land			34	0/1 (0 %)	0
Effective walkable area ratio			34	0/1 (0 %)	0
Connected node ratio			34	0/1 (0 %)	0
Herfindahl-hirschman index (diversity of land uses in the school neighbourhood to measure environmental supportiveness)			34	0/1 (0 %)	0
Streets are free from rubbish			34	0/1 (0 %)	0
Time					
Specific day of the week			54^b^, 54^g^	0/2 (0 %)	0
Time of the day (school time vs out of school time (reference))	54^b^, 54^g^	-		2/2 (100 %)	-
Policy variables: industry					
Advertisement			58	0/1 (0 %)	0
Policy variables: government					
Participation in healthy school programme			34, 34	0/2 (0 %)	0
Provision of PA information			34, 34	0/2 (0 %)	0
Provision of health promotion information	34	+	34	1/2 (50 %)	?
Provision of risks of unhealthy lifestyle information			34, 34	0/2 (0 %)	0
Hours of physical education	34	+		1/1 (100 %)	+
Extracurricular PA before school			34	0/1 (0 %)	0
Extracurricular PA during lunch breaks			34	0/1 (0 %)	0
Extracurricular PA during weekends	34	-		1/1 (100 %)	-
Duration of morning break (>15 minutes)	34	-		1/1 (100 %)	-
Duration of lunch break	34	-		1/1 (100 %)	-
Breaks: allowed to play outside in bad weather			34	0/1 (0 %)	0
Breaks: screenplay allowed			34	0/1 (0 %)	0
Breaks: >2 PA allowed			34	0/1 (0 %)	0
Existence of breakfast club			34	0/1 (0 %)	0
Existence of lollypop person (e.g. crossing guard)	34	-		1/1 (100 %)	-
Existence of park and stride			34	0/1 (0 %)	0
Existence of travel plan			34	0/1 (0 %)	0
Existence of walking bus			34	0/1 (0 %)	0
Provision of cycle training			34	0/1 (0 %)	0
Provision of pedestrian training	34	+		1/1 (100 %)	+

*SB* sedentary behaviour, *SES* socio-economic status

^1^Summary code is an overall summary of the findings for each variable separately

^2^ *n* = Number of analyses that support the direction of the association; *N* = number of analyses that have investigated and reported on possible associations between the variable and sedentary behaviour

^3^Shows the direction of the individual/summary association

Subgroup analyses: ^b^only in boys; ^g^only in girls; other subgroup analyses are listed but are not specified

**Table 4:** Determinants of subjectively measured total sedentary behaviour in children and direction and strength of association

	Related to sedentary behaviour		Unrelated to sedentary behaviour	Summary code^1^	
Variables	Reference number	Direction of association	Reference number	*n*/*N* for row (%)^2^	Association (+/−)^3^
Individual variables: biological/genetic					
Gender	33^b^	+	35, 63	1/3 (33 %)	0
Age (older)	33^b^, 33^g^, 35^b^, 35^g^, 52, 59^g^, 59^g^, 59^g^, 59^g^, 62^b^, 62^g^	+	63	11/12 (92 %)	++
Maturation	30^b,wk^, 62^b^, 62^g^, 62^b^, 62^g^	+	30^g,wk^, 30^b,wn^, 30^g,wn^	5/8 (63 %)	+
Weight status	28, 30^g,wn^	+	30^b,wn^, 30^b,wk^, 30^g,wk^, 62	2/6 (33 %)	0
SES (high)	62^g^	+	62^b^	1/2 (50 %)	?
Interpersonal variables: cultural					
Ethnicity (black)	33	+		1/1 (100 %)	+
Environmental variables					
Neighbourhood					
Neighbourhood SES (low)	33	+		1/1 (100 %)	+

*SES* socio-economic status

^1^Summary code is an overall summary of the findings for each variable separately

^2^ *n* = Number of analyses that support the direction of the association; *N* = number of analyses that have investigated and reported on possible associations between the variable and sedentary behaviour

^3^Shows the direction of the individual/summary association

Subgroup analyses: ^b^only in boys; ^g^only in girls; ^wk^only on weekdays; ^wn^only on weekend days; other subgroup analyses are listed but are not specified

**Table 5:** Determinants of subjectively measured screen time in children and direction and strength of association

	Related to screen time		Unrelated to screen time	Summary code^1^	
Variables	Reference number	Direction of association	Reference number	*n*/*N* for row (%)^2^	Association (+/−)^3^
Individual variables: biological/genetic					
Gender	35^b^, 60^b^	+	47^b^, 63	2/5 (40 %)	?
	47^g^	+			
Age (older)	15, 31^b^, 31^g^, 35^g^, 35, 40^b^, 40^g^, 40, 40, 40, 40, 43, 43, 43, 49^b^, 49^g^, 49, 49, 50^b^, 50^g^, 50^b^, 50^g^, 50, 50, 50, 50, 50, 50, 50, 50, 50, 50, 50, 50, 50, 50, 50, 52,	+	15, 35^b^, 41^g^, 43, 43, 43, 43, 48^b^, 50, 50, 50, 50, 50, 52, 59^g^, 63	43/62(69 %)	++
	59^g^, 59^g^, 60^b^, 62^b^, 62^g^	-			
	43, 48^g^, 60^g^				
Maturation	32^b^, 32	+	32^g^, 32, 32, 32	2/6 (33 %)	0
Weight status	53, 53	+		2/2 (100 %)	+
SES (high)	62^g^	+	62^b^	1/2 (50 %)	?
Individual variables: psychological/behavioural					
Depressive symptoms	48^b^, 48^g^, 51^g^	+	51^b^	3/4 (75 %)	+
SB at baseline	40, 49, 50	+		3/3 (100 %)	+
Eating in front of TV	49	+		1/1 (100 %)	+
Food intake	53 (med)	+		1/1 (100 %)	+
Perceived academic rank	48^b^	+		1/2 (50 %)	?
	48^g^	-			
Academic expectation	48^b^	+	48^g^	1/2 (50 %)	?
Future expectations	48^b^	-	48^g^	1/2 (50 %)	?
Value of health, achievement and appearance	48^g^	-	48^b^	1/2 (50 %)	?
Spiritual beliefs	48^b^	-	48^g^	1/2 (50 %)	?
Interpersonal variables: cultural					
Ethnicity (African-American)	48^b^, 48^g^	+		2/2 (100 %)	+
Interpersonal variables: social					
Family influences					
Mother at home			48^b^, 48^g^	0/2 (0 %)	0
Father at home			48^b^, 48^g^	0/2 (0 %)	0
Maternal education			49	0/1 (0 %)	0
Parents working full time			48^b^, 48^g^	0/2 (0 %)	0
Parental education	48^g^	-	48^b^	1/2 (50 %)	?
Parental weight status			41^g^	0/1 (0 %)	0
Parental behaviour					
Child’s perception of mother or father caring about staying fit			44, 44, 44, 44, 44, 44, 44, 44	0/8 (0 %)	0
Child’s perception of maternal or paternal encouragements to be active	44, 44, 44	-	44, 44, 44, 44, 44	3/8 (38 %)	?
Maternal TV viewing time			42^g^	0/1 (0 %)	0
Paternal TV viewing time			42^g^, 42^b^	0/2 (0 %)	0
Parents’ use of TV as recreation			42^g^	0/1 (0 %)	0
Number of TV-related parenting risk factors (e.g. high maternal TV viewing)	42^g^	+		1/1 (100 %)	+
Family behaviour					
Watching TV as a family	42^g^	+		1/1 (100 %)	+
Rules and restrictions					
Maternal authority	48^g^	-	48^b^	1/2 (50 %)	?
Paternal authority			48^b^, 48^g^	0/2 (0 %)	0
Parental perceptions					
Parents believe there is a high crime rate in their neighbourhood			52, 52	0/2 (0 %)	0
Parents consider stranger danger to be a concern			52, 52	0/2 (0 %)	0
Social network					
Social network score			52, 52	0/2 (0 %)	0
Social trust and cohesion score			52, 52	0/2 (0 %)	0
Environmental variables: micro					
Home					
Number of TVs at home			49	0/1 (0 %)	0
Video cassette recorder at home			49	0/1 (0 %)	0
Active games instead of traditional electronic games	56, 56	-	56, 56	2/4 (50 %)	?
Removal of traditional electronic games	56, 56	-	56, 56	2/4 (50 %)	?
Electronic equipment in the bedroom			38, 38	0/2 (0 %)	0
Computer in the bedroom	38	-	38, 38	1/3 (33 %)	0
TV in the bedroom	31^b^, 38, 49	+	31^g^, 38, 38	3/6 (50 %)	?
Neighbourhood					
Living in a cul-de-sac	52	-	52	1/2 (50 %)	?
Parents are satisfied with quality of parks and playgrounds in their neighbourhood	52	-	52	1/2 (50 %)	?
Distance to closest public open space from home			52, 52	0/2 (0 %)	0
Closest park: area of closest park to home			52, 52	0/2 (0 %)	0
Closest park: number of recreational facilities			52, 52	0/2 (0 %)	0
Closest park: number of playgrounds			52, 52	0/2 (0 %)	0
Closest park: number of amenities			52, 52	0/2 (0 %)	0
Closest park: walking paths	52	+	52	1/2 (50 %)	?
Closest park: cycling paths			52, 52	0/2 (0 %)	0
Closest park: lighting along paths			52, 52	0/2 (0 %)	0
Closest park: trees providing shade			52, 52	0/2 (0 %)	0
Closest park: water feature			52, 52	0/2 (0 %)	0
Closest park: signage regarding dogs			52, 52	0/2 (0 %)	0
Time					
Time (year)	43, 43, 61^b^, 61^g^, 61, 61, 61, 61, 61, 61	+	43, 43	10/12 (83 %)	+

*SB* sedentary behaviour, *SES* socio-economic status

^1^Summary code is an overall summary of the findings for each variable separately

^2^ *n* = Number of analyses that support the direction of the association; *N* = number of analyses that have investigated and reported on possible associations between the variable and sedentary behaviour

^3^Shows the direction of the individual/summary association

Subgroup analyses: ^b^only in boys; ^g^only in girls; other subgroup analyses are listed but are not specified

### Individual determinants

#### Biological/genetic

##### Age

Eleven studies investigated the association between age and total SB [15, 33, 35, 36, 39, 52, 54, 57, 59, 62, 63]. Five studies [15, 36, 39, 54, 57] were based on objectively measured total SB and six studies [33, 35, 52, 59, 62, 63] were based on subjectively measured total SB. In both cases (i.e., objectively [36, 39, 54, 57] and subjectively [33, 35, 52, 59, 62]) there is evidence for a significant association with youth engaging more in total sedentary time when they grow older, leading to consistent evidence for age as a determinant of sedentary time [33, 35, 36, 39, 52, 54, 57, 59, 62]. Also for screen time there was a consistent association with age with youth engaging in more screen time when they grow older [15, 31, 35, 40, 41, 43, 48–50, 52, 59, 60, 62, 63].

##### Gender

The association between gender and SB was examined in four studies [33, 35, 54, 63]. One study [54] was based on objectively measured total SB and showed that there is evidence for a consistent association between gender and objectively measured total SB with boys engaging in less total SB compared to girls. Furthermore, no evidence was found for the association between gender and subjectively measured total SB. Based on those studies, no evidence for an association was reported. There was inconsistent evidence for an association between gender and screen time [35, 47, 60, 63].

##### Weight status

Three studies examined the association between weight status and subjectively measured total SB, but found no evidence of an association [28, 30, 62]. On the other hand, there is evidence of an association with screen time, with heavier youth engaging in higher levels of screen time over time [53].

##### Socioeconomic status

Two studies considered the association between socioeconomic status (SES) and total SB [39, 62]. Children from families with a higher SES engaged in higher amounts of objectively measured SB [39]. However, there is inconsistent evidence for the association between SES and subjectively measured SB [62]. Also for screen time specifically, inconsistent evidence was found for the association with SES [62].

### Psychological/behavioural

Baseline assessment of screen time was found to be significantly associated with screen time at follow-up [40, 49, 50], indicating tracking of screen time over time. Scoring high on depressive symptoms was found to be significantly associated with screen time behaviour [48, 51]. Youth with more depressive symptoms tend to spend more time in front of screens. Furthermore, there is evidence for the association between eating in front of TV and screen time, with eating more frequently in front of TV being associated with more screen time [49].

### Interpersonal determinants

#### Cultural

There is evidence that being black is associated with more subjectively measured total SB [33]. In addition, African-Americans engaged in more screen time [48].

#### Social

There is inconsistent evidence or no evidence for the associations for most social determinants (e.g. parental education, number of siblings, maternal PA). Only the association between ostracism (absence of social support) and objectively measured total SB [46], the association between number of TV related parenting risk factors and screen time [42], and the association between watching TV as a family and screen time [42] were significant. The absence of social support can increase children’s time spent sedentary [46] and having more TV related parenting risk factors and watching more TV as a family, can result in higher screen time in youth.

### Environmental determinants

For most environmental determinants (e.g. electronic games at home, living in a cul-de-sac, playground area at school) there is no evidence or inconsistent evidence for an association. However, youth living in lower SES neighbourhoods engaged in more subjectively measured total SB [33]. There is evidence for the association between playground density and objectively measured total SB, with more children sharing a playground resulting in higher levels of SB [37]. In addition, there is evidence for a consistent association between availability of play and sports equipment and changing facilities with higher objectively measured total SB [34]. The existence of safe places to cross roads near the school, was associated with lower levels of objectively measured total SB [34].

Youth spent less time on objectively measured SB during school hours compared to out of school time [54]. There is evidence for a consistent association between screen time and year of measurement which indicates an increase in screen time over time [43, 61].

### Policy level determinants

#### Governmental

Unexpectedly, more hours of physical education and the provision of pedestrian training were associated with a higher total sedentary time [34]. Furthermore, having a crossing guard to help children cross the roads near school safely, having more extracurricular PA during weekends and having longer lunch breaks resulted in less time spent sedentary [34].

## Discussion

The current paper reviewed the determinants of SB in toddlers, preschoolers, children and adolescents. SB research is a relatively new field, which is reflected in the fact that more than half of the included studies were published from 2010 onwards. In addition, most studies were conducted in Europe, USA, and Australia, which shows a wide international spread of studies, but largely restricted to high income countries. Also in the review of Uijtdewilligen et al. (2011), 28 of the 30 included articles were carried out in high income countries (USA, Canada, Great Britain, Australia, France, The Netherlands, Estonia, Sweden and New Zealand) [21]. This shows the need for more research in low and middle income countries as information from those countries is currently missing. The current review took a stringent approach by including only studies with a longitudinal design in order to provide evidence on prediction rather than mere association. However, only a few studies looked at a comprehensive set of factors at various levels, and as a consequence, the evidence available on the identified determinants is largely derived from only one or two studies. Nevertheless, these studies were in general of high quality.

In general, screen time – and TV viewing in particular – is the most commonly measured SB in youth and is frequently used as a proxy marker of total SB [64]. However, the results of the current systematic literature review clearly show that the determinants of total SB (e.g. maturation, SES, playground density) differ from the determinants of screen time (e.g. weight status, eating in front of TV, watching TV as a family). Also within the nine studies that looked simultaneously at screen time and total sedentary time, we see that for the majority of investigated determinants, there are differences in significance between sedentary time and screen time [15, 35, 38, 51, 52, 56, 59, 62, 63]. Similarly, Verloigne et al. (2013) reported that TV and computer time do not adequately reflect total SB in European 10–12 year old children [11]. Consequently, solely focussing on the determinants of screen time may be too limited to obtain meaningful changes in total SB, as only one type of SB is then targeted. However, since looking at “contextual” indicators of SB (such as screen time) often gives useful information regarding potential preventive strategies, future studies should look at both outcomes.

All three studies examining tracking of screen time found that baseline assessment of screen time was significantly associated with screen time at follow-up [40, 49, 50]. Also the review of Biddle et al. (2010) showed that there is evidence for tracking of children’s SB from childhood into adolescence and adulthood [65]. Therefore, intervening in early age may be an effective strategy [66]. Future interventions aimed at decreasing sedentary behaviours should target young children before sedentary behaviours become entrenched into living habits. However, preventive interventions should be considered at all ages since it may still be possible to change behaviours at later ages. Furthermore, the lack of studies in this review investigating determinants of SB in toddlers and pre-schoolers [63], should be noted.

The majority of the identified determinants of both total SB and screen time, were found at the individual level of the socio-ecological model [20] (e.g., age, maturation, weight status, SES). The review by Uijtdewilligen et al. (2011) which at first found insufficient evidence for determinants of sedentary behaviour, only found strong evidence for a positive association between BMI and child sedentary behaviour after conducting a sensitivity test (taking into account the high quality studies twice and low quality studies once) [21]. However, it is difficult or even not possible to modify these individual determinants. Therefore, when developing interventions to reduce SB, differences in age, maturation, weight status and SES should be kept in mind.

In relation to environmental determinants, it firstly has to be acknowledged that although some studies examined a very large number of neighbourhood and school variables [34, 39, 45, 52], hardly any were found to be associated with total SB or screen time. However, the home and the school environment are important settings in which children and adolescents spend most of their waking time. In the home environment, there was no evidence for an association between the number of TVs and having a TV in the bedroom with screen time although this might be due to the fact that recently in many households mobile phones or tablets became an important alternative to TV screens. However, there was evidence for a positive association between eating in front of TV with more screen time. This phenomenon, called ‘constant television households’, which means that the TV is on during meals, promotes more overall children’s TV watching and could be an important target to decrease screen time [67].

The results from one study included in this review suggest that at the school level, lowering the playground density could be an effective intervention for decreasing children’s sedentary time [37]. Although the consistent intervention effects were rather small, decreasing the playground density by splitting up the recesses of different groups of children and decreasing the number of children sharing the playground, could be effective in a larger multi-component school-based intervention to decrease sedentary time. Since this simple and sustainable strategy is free of costs, requires no teacher training or alterations to the facilities, and does not put extra pressure on the curriculum, it merits further attention in improving sedentary levels in both younger and older children.

Counter-intuitively, one study showed a consistent association between availability of play and sports equipment with higher total SB [34]. However, it should be noted that there was no distinction between different kinds of equipment. Different kinds of equipment might stimulate youth to be more active (e.g. availability of balls) but it might induce more SB in other children because children who use this equipment may dominate the playground which can cause the other children perceiving the environment as more dangerous or too crowded to play safely.

In addition, it might be possible that there is too much equipment available at the school, which makes it a burden for youth to use. A further possibility might be that the equipment is heavy or too complex and requires expertise and organisation to use. Finally, some play or sports equipment might also stimulate SB, for example the provision of little toys to use in the sandbox. Therefore, in order to reduce SB it may be important to give careful consideration to the specific play and sports equipment provided. Older children might not be challenged by play equipment which is meant for younger children [68] or vice versa. It might also be advisable to create certain zones for ball games.

The results of the current systematic review suggest that if there are safe places to cross roads near the school and a crossing guard is present, less SB in children is noticed [34]. Safety is known to be the main factor for the decision making in transport mode in youth [69, 70]. Consequently, it can be assumed that safe cross roads cause less passive transport to school (e.g., by car, by bus). This underlines the importance of traffic safety issues near the school (e.g., design and accessibility of safe places to cross roads near schools, the provision of crossing guards).

Finally, some policy determinants showed a consistent association with total SB. These determinants are mainly found at the school policy level (e.g., hours of physical education, duration of morning break (>15 min) and lunch break). More hours of physical education induced higher levels of total SB in primary schoolchildren. A possible explanation might be the fact that children might be more tired after a physical education lesson, and thus compensate for example during recess [71]. Furthermore, other studies in secondary schools already found that physical education lesson are largely sedentary [72–75]. As school environment and school policy were identified as important determinants of SB, in school principals and teachers, the awareness of the importance of decreasing children’s SB should be raised.

### Strengths and limitations

A first strength of this systematic review is that the included studies comprised a wide range of sample sizes. However, a median sample size of 759 participants across the included studies, strengthens the generalizability of our results. A second strength is the use of a high quality standardized protocol and data-extraction process. The evidence from the included studies seems trustworthy as it generally comes from high quality studies (median: 82 %). However, the level of evidence may be somewhat affected by study methodology. For example, in the younger age groups (toddlers and pre-schoolers and primary schoolchildren), proxy reported questionnaires were sometimes used to assess children’s SB as young children cannot self-report on their levels of SB because of their cognitive limitations. Therefore, parents often report on their child’s SB but recalling young children’s SB might be difficult for them [76]. For older children like adolescents, sometimes self-report questionnaires were used, which may have led to social desirability bias.

Furthermore, the used quality assessment tool did not assess losses of follow-up. Another limitation is that the systematic literature search was conducted one year ago so as a result more recently published studies were not included in this review. Finally, in the current review multi-component interventions were excluded. However, significant associations found in the included single-component interventions, enable researchers to specifically focus on those determinants in future interventions.

## Conclusions

In conclusion, while the research on SB has only recently emerged, results of this systematic literature review show that several longitudinal studies have been carried out looking into the determinants of SB in youth. Not only individual but also interpersonal, environmental and policy determinants according to socioecological models have been studied. As SB tends to increase with age, interventions should start in young children. Furthermore, there is consistent evidence for weight status and baseline assessment of screen time being positively associated with screen time (at follow-up). A higher playground density and a higher availability of play and sports equipment at school, were consistently related to an increased total SB. Evidence was also reported for the presence of safe places to cross roads and lengthening morning and lunch breaks being associated with less total SB. However, most factors were examined in only one or two studies and few studies examined a comprehensive set of factors at different levels of influences. Furthermore, the inconclusive results of the present review highlight the need for more longitudinal research and well-designed randomized controlled experiments.

## Additional files

Additional file 1:
**Details of search strategy: Four main elements of the search and complete list of search terms.** (DOCX 15 kb) Additional file 2:
**Quality Assessment of quantitative studies.** (DOCX 15 kb)

## Abbreviations

CPM: Counts per minute
DEDIPAC: DEterminants of DIet and Physical ACtivity
SB: Sedentary behaviour
SES: Socioeconomic status

## References

[1] Chinapaw M, Altenburg T, Brug J (2015). Sedentary behaviour and health in children - evaluating the evidence. Prev Med.

[2] Carrel AL, Clark RR, Peterson SE, Nemeth BA, Sullivan J, Allen DB (2005). Improvement of fitness, body composition, and insulin sensitivity in overweight children in a school-based exercise program: a randomized, controlled study. Arch Pediatr Adolesc Med.

[3] Gortmaker SL, Peterson K, Wiecha J, Sobol AM, Dixit S, Fox MK (1999). Reducing obesity via a school-based interdisciplinary intervention among youth: Planet Health. Arch Pediatr Adolesc Med.

[4] Robinson TN (1999). Reducing children’s television viewing to prevent obesity: a randomized controlled trial. JAMA.

[5] Chinapaw MJ, Proper KI, Brug J, van Mechelen W, Singh AS (2011). Relationship between young peoples’ sedentary behaviour and biomedical health indicators: a systematic review of prospective studies. Obes Rev.

[6] Tremblay MS, LeBlanc AG, Kho ME, Saunders TJ, Larouche R, Colley RC (2011). Systematic review of sedentary behaviour and health indicators in school-aged children and youth. Int J Behav Nutr Phys Act.

[7] Ekelund U, Luan J, Sherar LB, Esliger DW, Griew P, Cooper A (2012). Moderate to vigorous physical activity and sedentary time and cardiometabolic risk factors in children and adolescents. JAMA.

[8] Herman KM, Hopman WM, Sabiston CM (2015). Physical activity, screen time and self-rated health and mental health in Canadian adolescents. Prev Med.

[9] Gracia-Marco L, Rey-Lopez JP, Santaliestra-Pasias AM, Jimenez-Pavon D, Diaz LE, Moreno LA (2012). Sedentary behaviours and its association with bone mass in adolescents: the HELENA Cross-Sectional Study. BMC Public Health.

[10] Chastin SF, Mandrichenko O, Skelton DA (2014). The frequency of osteogenic activities and the pattern of intermittence between periods of physical activity and sedentary behaviour affects bone mineral content: the cross-sectional NHANES study. BMC Public Health.

[11] Verloigne M, Van LW, Maes L, Yildirim M, Chinapaw M, Manios Y (2013). Self-reported TV and computer time do not represent accelerometer-derived total sedentary time in 10 to 12-year-olds. Eur J Pub Health.

[12] Brug J, van Stralen MM, Te Velde SJ, Chinapaw MJ, De Bourdeaudhuij I, Lien N (2012). Differences in Weight Status and Energy-Balance Related Behaviors among Schoolchildren across Europe: The ENERGY-Project. PLoS One.

[13] Tremblay MS, LeBlanc AG, Janssen I, Kho ME, Hicks A, Murumets K (2011). Canadian sedentary behaviour guidelines for children and youth. Appl Physiol Nutr Metab.

[14] Salmon J, Tremblay MS, Marshall SJ, Hume C (2011). Health risks, correlates, and interventions to reduce sedentary behavior in young people. Am J Prev Med.

[15] Janz KF, Burns TL, Levy SM (2005). Tracking of activity and sedentary behaviors in childhood: the Iowa Bone Development Study. Am J Prev Med.

[16] Hirvensalo M, Lintunen T (2011). Life-course perspective for physical activity and sports participation. Eur Rev Aging Phys Act.

[17] Owen N, Sugiyama T, Eakin EE, Gardiner PA, Tremblay MS, Sallis JF (2011). Adults’ sedentary behavior determinants and interventions. Am J Prev Med.

[18] van Sluijs EM, McMinn AM, Griffin SJ (2007). Effectiveness of interventions to promote physical activity in children and adolescents: systematic review of controlled trials. BMJ.

[19] van Sluijs EM, Kriemler S, McMinn AM (2011). The effect of community and family interventions on young people’s physical activity levels: a review of reviews and updated systematic review. Br J Sports Med.

[20] Sallis JF, Owen N, Fisher EB, Glanz K, Rimer BK, Viswanath K (2008). Ecological models of health behavior. Health behavior and health education. Theory, research, and practice.

[21] Uijtdewilligen L, Nauta J, Singh AS, van Mechelen W, Twisk JW, van der Horst K (2011). Determinants of physical activity and sedentary behaviour in young people: a review and quality synthesis of prospective studies. Br J Sports Med.

[22] Lakerveld J, van der Ploeg HP, Kroeze W, Ahrens W, Allais O, Andersen LF (2014). Towards the integration and development of a cross-European research network and infrastructure: the DEterminants of DIet and Physical ACtivity (DEDIPAC) Knowledge Hub. Int J Behav Nutr Phys Act.

[23] Bauman AE, Sallis JF, Dzewaltowski DA, Owen N (2002). Toward a better understanding of the influences on physical activity: the role of determinants, correlates, causal variables, mediators, moderators, and confounders. Am J Prev Med.

[24] Treuth MS, Schmitz K, Catellier DJ, McMurray RG, Murray DM, Almeida MJ (2004). Defining accelerometer thresholds for activity intensities in adolescent girls. Med Sci Sports Exerc.

[25] Centre for Reviews and Dissemination: *Systematic Reviews: CRD’s guidance for undertaking systematic reviews in health care*. York: CRD, University of York; 2009.

[26] Sallis JF, Prochaska JJ, Taylor WC (2000). A review of correlates of physical activity of children and adolescents. Med Sci Sports Exerc.

[27] Kmet L, Lee RC, Cook LS. Standard quality assessment criteria for evaluating primary research papers from a variety of fields**.** Institute of Health Economics 2004;1–31.

[28] Hjorth MF, Chaput JP, Ritz C, Dalskov SM, Andersen R, Astrup A (2014). Fatness predicts decreased physical activity and increased sedentary time, but not vice versa: support from a longitudinal study in 8- to 11-year-old children. Int J Obes (Lond).

[29] Ridgway CL, Brage S, Sharp SJ, Corder K, Westgate KL, van Sluijs EM (2011). Does birth weight influence physical activity in youth? A combined analysis of four studies using objectively measured physical activity. PLoS One.

[30] Murdey ID, Cameron N, Biddle SJ, Marshall SJ, Gorely T (2005). Short-term changes in sedentary behaviour during adolescence: Project STIL (Sedentary Teenagers and Inactive Lifestyles). Ann Hum Biol.

[31] Delmas C, Platat C, Schweitzer B, Wagner A, Oujaa M, Simon C (2007). Association between television in bedroom and adiposity throughout adolescence. Obesity (Silver Spring).

[32] van Jaarsveld CH, Fidler JA, Simon AE, Wardle J (2007). Persistent impact of pubertal timing on trends in smoking, food choice, activity, and stress in adolescence. Psychosom Med.

[33] Brodersen NH, Steptoe A, Boniface DR, Wardle J (2007). Trends in physical activity and sedentary behaviour in adolescence: ethnic and socioeconomic differences. Br J Sports Med.

[34] Mantjes JA, Jones AP, Corder K, Jones NR, Harrison F, Griffin SJ (2012). School related factors and 1 yr change in physical activity amongst 9–11 year old English schoolchildren. Int J Behav Nutr Phys Act.

[35] Wickel EE, Issartel J, Belton S (2013). Longitudinal change in active and sedentary behavior during the after-school hour. J Phys Act Health.

[36] Ortega FB, Konstabel K, Pasquali E, Ruiz JR, Hurtig-Wennlof A, Maestu J (2013). Objectively measured physical activity and sedentary time during childhood, adolescence and young adulthood: a cohort study. PLoS One.

[37] D’Haese S, Van Dyck D, De Bourdeaudhuij I, Cardon G (2013). Effectiveness and feasibility of lowering playground density during recess to promote physical activity and decrease sedentary time at primary school. BMC Public Health.

[38] Atkin AJ, Corder K, van Sluijs EM (2013). Bedroom media, sedentary time and screen-time in children: a longitudinal analysis. Int J Behav Nutr Phys Act.

[39] Atkin AJ, Corder K, Ekelund U, Wijndaele K, Griffin SJ, van Sluijs EM (2013). Determinants of change in children’s sedentary time. PLoS One.

[40] Raudsepp L, Neissaar I, Kull M (2008). Longitudinal stability of sedentary behaviors and physical activity during early adolescence. Pediatr Exerc Sci.

[41] Treuth MS, Butte NF, Adolph AL, Puyau MR (2004). A longitudinal study of fitness and activity in girls predisposed to obesity. Med Sci Sports Exerc.

[42] Davison KK, Francis LA, Birch LL (2005). Links between parents’ and girls’ television viewing behaviors: a longitudinal examination. J Pediatr.

[43] Nelson MC, Neumark-Stzainer D, Hannan PJ, Sirard JR, Story M (2006). Longitudinal and secular trends in physical activity and sedentary behavior during adolescence. Pediatrics.

[44] Bauer KW, Nelson MC, Boutelle KN, Neumark-Sztainer D (2008). Parental influences on adolescents’ physical activity and sedentary behavior: longitudinal findings from Project EAT-II. Int J Behav Nutr Phys Act.

[45] Evenson KR, Murray DM, Birnbaum AS, Cohen DA (2010). Examination of perceived neighborhood characteristics and transportation on changes in physical activity and sedentary behavior: The Trial of Activity in Adolescent Girls. Health Place.

[46] Barkley JE, Salvy SJ, Roemmich JN (2012). The effect of simulated ostracism on physical activity behavior in children. Pediatrics.

[47] Datar A, Nicosia N, Shier V (2013). Parent perceptions of neighborhood safety and children’s physical activity, sedentary behavior, and obesity: evidence from a national longitudinal study. Am J Epidemiol.

[48] Schmitz KH, Lytle LA, Phillips GA, Murray DM, Birnbaum AS, Kubik MY (2002). Psychosocial correlates of physical activity and sedentary leisure habits in young adolescents: The teens eating for energy and nutrition at school study. Prev Med.

[49] Saelens BE, Sallis JF, Nader PR, Broyles SL, Berry CC, Taras HL (2002). Home environmental influences on children’s television watching from early to middle childhood. J Dev Behav Pediatr.

[50] Francis SL, Stancel MJ, Sernulka-George FD, Broffitt B, Levy SM, Janz KF (2011). Tracking of TV and video gaming during childhood: Iowa bone development study. Int J Behav Nutr Phys Act.

[51] Hume C, Timperio A, Veitch J, Salmon J, Crawford D, Ball K (2011). Physical activity, sedentary behavior, and depressive symptoms among adolescents. J Phys Act Health.

[52] Veitch J, Timperio A, Crawford D, Abbott G, Giles-Corti B, Salmon J (2011). Is the neighbourhood environment associated with sedentary behaviour outside of school hours among children?. Ann Behav Med.

[53] Fuller-Tyszkiewicz M, Skouteris H, Hardy LL, Halse C (2012). The associations between TV viewing, food intake, and BMI. A prospective analysis of data from the Longitudinal Study of Australian Children. Appetite.

[54] Telford RM, Telford RD, Cunningham RB, Cochrane T, Davey R, Waddington G (2013). Longitudinal patterns of physical activity in children aged 8 to 12 years: the LOOK study. Int J Behav Nutr Phys Act.

[55] Ridgers ND, Timperio A, Crawford D, Salmon J (2013). What factors are associated with adolescents’ school break time physical activity and sedentary time?. PLoS One.

[56] Straker LM, Abbott RA, Smith AJ (2013). To remove or to replace traditional electronic games? A crossover randomised controlled trial on the impact of removing or replacing home access to electronic games on physical activity and sedentary behaviour in children aged 10–12 years. BMJ Open.

[57] Arundell L, Ridgers ND, Veitch J, Salmon J, Hinkley T, Timperio A (2013). 5-year changes in afterschool physical activity and sedentary behavior. Am J Prev Med.

[58] Atlantis E, Salmon J, Bauman A (2008). Acute effects of advertisements on children’s choices, preferences, and ratings of liking for physical activities and sedentary behaviours: a randomised controlled pilot study. J Sci Med Sport.

[59] Hardy LL, Bass SL, Booth ML (2007). Changes in sedentary behavior among adolescent girls: a 2.5-year prospective cohort study. J Adolesc Health.

[60] Ziviani J, Macdonald D, Ward H, Jenkins D, Rodger S (2008). Physical activity of young children: a two-year follow-up. Phys Occup Ther Pediatr.

[61] Cui Z, Hardy LL, Dibley MJ, Bauman A (2011). Temporal trends and recent correlates in sedentary behaviours in Chinese children. Int J Behav Nutr Phys Act.

[62] Trang NH, Hong TK, van der Ploeg HP, Hardy LL, Kelly PJ, Dibley MJ (2013). Longitudinal sedentary behavior changes in adolescents in Ho Chi Minh City. Am J Prev Med.

[63] Taylor RW, Murdoch L, Carter P, Gerrard DF, Williams SM, Taylor BJ (2009). Longitudinal study of physical activity and inactivity in preschoolers: the FLAME study. Med Sci Sports Exerc.

[64] Atkin AJ, Gorely T, Clemes SA, Yates T, Edwardson C, Brage S (2012). Methods of measurement in epidemiology: sedentary behaviour. Int J Epidemiol.

[65] Biddle SJ, Pearson N, Ross GM, Braithwaite R (2010). Tracking of sedentary behaviours of young people: a systematic review. Prev Med.

[66] Sonneville KR, La Pelle N, Taveras EM, Gillman MW, Prosser LA (2009). Economic and other barriers to adopting recommendations to prevent childhood obesity: results of a focus group study with parents. BMC Pediatr.

[67] Medrich EA (1979). Constant television: A background to daily life. J Commun.

[68] Veitch J, Bagley S, Ball K, Salmon J (2006). Where do children usually play? A qualitative study of parents’ perceptions of influences on children’s active free-play. Health Place.

[69] Panter JR, Jones AP, van Sluijs EM (2008). Environmental determinants of active travel in youth: a review and framework for future research. Int J Behav Nutr Phys Act.

[70] Ducheyne F, De BI, Spittaels H, Cardon G (2012). Individual, social and physical environmental correlates of ‘never’ and ‘always’ cycling to school among 10 to 12 year old children living within a 3.0 km distance from school. Int J Behav Nutr Phys Act.

[71] Ridgers ND, Timperio A, Cerin E, Salmon J (2014). Compensation of physical activity and sedentary time in primary school children. Med Sci Sports Exerc.

[72] Dudley DA, Okely AD, Cotton WG, Pearson P, Caputi P (2012). Physical activity levels and movement skill instruction in secondary school physical education. J Sci Med Sport.

[73] McKenzie TL, Catellier DJ, Conway T, Lytle LA, Grieser M, Webber LA (2006). Girls’ activity levels and lesson contexts in middle school PE: TAAG baseline. Med Sci Sports Exerc.

[74] Chow BC, McKenzie TL, Louie L (2015). Physical activity and environmental influences during secondary school physical education. J Teach Phys Educ.

[75] Cardon G, Verstraete S, De Bourdeaudhuij I, De Clercq D (2004). Physical activity levels during elementary physical education in Flanders: swimming classes compared to regular classes. J Teach Phys Educ.

[76] Corder K, van Sluijs EM, Wright A, Whincup P, Wareham NJ, Ekelund U (2009). Is it possible to assess free-living physical activity and energy expenditure in young people by self-report?. Am J Clin Nutr.

