# Supplementary material for: A systematic review of determinants of sedentary behaviour in youth: a DEDIPAC-study
Source: Int J Behav Nutr Phys Act. 2015 Oct 9;12:133. doi: 10.1186/s12966-015-0291-4 (PMC4600309; doi:10.1186/s12966-015-0291-4)
Supplement: Additional file 1: — Details of search strategy: Four main elements of the search and complete list of search terms. (DOCX 15 kb) [file 12966_2015_291_MOESM1_ESM.docx]

# Additional File 1 – Details of search strategy: Four main elements of the search and complete list of search terms

| Sedentary behaviour and its synonyms | ("sedentary behaviour" or "sedentary behavior" or "sedentary time" or "sedentariness" or "sedentary lifestyle" or “physical inactivity”) |
| --- | --- |
| Determinants and its synonyms | (“determinants or correlates or mediators or contributors or association or modifiers or confounders or patterns”) |
| Types of sedentary behaviour | ("television watching" or "television viewing" or "video viewing" or "screen time" or "TV" or "video games" or "electronic game playing" or "computer gaming" or "computer time" or "computer use" or "media time" or "media use" or "web browsing" or "screen time" or "bedroom media" or "electronic media" or "PC" or "PC use" or "occupational sitting" or "deskbound" or "reading" or "motor* transport" ) |
| Possible determinants of sedentary behaviour | (“age or gender or body mass index or weight status or education or income or marital status or ethnicity or "genetic factors" or pain or fatigue or mobility or stiffness or "chronic disease" or “co-morbidities” or “physical function” or disability or impairment)  (“motivational or intention or impulsiveness or "cognitive ability" or temperament or attitude or attitudes or "perceived behavioural control" or "perceived physical condition" or planning or "self- efficacy" or "self-esteem" or personality or knowledge or beliefs or "physical activity levels" or "past physical activity levels" or "past sedentary behaviours" or "past sedentary behaviors" or "past sedentariness" or smoking or "family support" or "peer support" or "social support" or "perceived peer attitudes" or "social isolation")  (“aesthetics or "accessibility of facilities" or "convenience of facilities" or "availability of recreational space" or "availability of pathways" or "availability of footpaths" or "availability of sidewalks" or "convenience of public transport" or streetlights or "traffic volume" or "traffic safety" or "winter season" or "bad weather" or "air pollution" or "coastal location" or "noise pollution" or "urban sprawl" or "urbanisation" or “walkability” or “environment”) |
