# Supplementary material for: A systematic review of determinants of sedentary behaviour in youth: a DEDIPAC-study
Source: Int J Behav Nutr Phys Act. 2015 Oct 9;12:133. doi: 10.1186/s12966-015-0291-4 (PMC4600309; doi:10.1186/s12966-015-0291-4)
Supplement: Additional file 2: — Quality Assessment of quantitative studies. (DOCX 15 kb) [file 12966_2015_291_MOESM2_ESM.docx]

# Additional File 2 – Quality Assessment of quantitative studies

Checklist for assessing the quality of quantitative studies

| **Criteria** | | **Yes (2)** | **Partial (1)** | **No (0)** | **N/A** |
| --- | --- | --- | --- | --- | --- |
| 1 | Question / objective sufficiently described? |  |  |  |  |
| 2 | Study design evident and appropriate? |  |  |  |  |
| 3 | Method of subject/comparison group selection or source of information/input variables described and appropriate? |  |  |  |  |
| 4 | Subject and comparison group (if applicable) characteristics sufficiently described? |  |  |  |  |
| 5 | If interventional and random allocation was possible, was it reported? |  |  |  |  |
| 6 | If interventional and blinding of investigators was possible, was it reported? |  |  |  |  |
| 7 | If interventional and blinding of subjects was possible, was it reported? |  |  |  |  |
| 8 | Outcome and (if applicable) exposure measure(s) well defined and robust to measurement / misclassification bias? Means of assessment reported? |  |  |  |  |
| 9 | Sample size appropriate? |  |  |  |  |
| 10 | Analytic methods described/justified and appropriate? |  |  |  |  |
| 11 | Some estimate of variance is reported for the main results? |  |  |  |  |
| 12 | Controlling for confounding? |  |  |  |  |
| 13 | Results reported in sufficient detail? |  |  |  |  |
| 14 | Conclusion supported by the results? |  |  |  |  |

**SCORING PROCESS**

Each question can be answered with “yes”, “partial”, “no” or “not applicable”.

The summary score is the total sum:

((number of “yes” x2) + (number of “partial” x1) / total possible sum (28 – (number of “not applicable” x2)
